# Impact of community-based interventions on condom use in the Tłįchǫ region of Northwest Territories, Canada

**DOI:** 10.1186/1472-6963-11-S2-S9

**Published:** 2011-12-21

**Authors:** Karen E Edwards, Nancy Gibson, Jim Martin, Steven Mitchell, Neil Andersson

**Affiliations:** 1 Universidad Autónoma de Guerrero, Mexico; 2 CIETcanada, Ottawa, Canada; 3 Tłįchǫ Community Services Agency, Canada; 4 Centro de Investigación de Enfermedades Tropicales (CIET), Universidad Autónoma de Guerrero, Mexico

**Keywords:** Tlicho (Tłįchǫ), social audit, Northwest Territories, sexually transmitted infections, condom use

## Abstract

**Background:**

Since 2005, the Tłįchǫ Community Services Agency (TCSA) in Canada's Northwest Territories (NT) has addressed rising rates of sexually transmitted infections (STI). In 2009, STI rates in the NT were ten times higher than the national rate and Tłįchǫ regional rates were nearly four times that of the NT – 91 cases per 1000 people. We describe a social audit process that assessed the impact of an evidence-based community-led intervention.

**Methods:**

A baseline survey of sexual health knowledge, attitudes and behaviours in 2006/07 provided evidence for a Community Action Research Team (CART) to develop and to put in place culturally appropriate interventions in the Tłįchǫ region. A follow-up study in 2010 sought to assess the impact of CART activities on condom use and underlying conscious knowledge, attitudes, subjective norms, intention to change, sense of agency and discussions related to condom use and STI risks. We report the contrasts using Odds Ratios (OR) and 95% confidence intervals (CI).

**Results:**

One in every three follow-up respondents (315/808) participated in at least one CART activity. Participation in highly ranked interventions was associated with increased condom use during the last sexual encounter (OR 1.45, 95%CI 1.07-1.98). Those exposed to three or more activities were more likely to talk openly about condoms (OR 2.08, 95%CI 1.41-3.28), but were also less likely to be monogamous (OR 0.49, 95%CI 0.29-0.90).

**Conclusions:**

The measurable impact on condom use indicates a strong beginning for the Tłįchǫ community intervention programmes. The interventions also seem to generate increased discussion, often a precursor to action. The Tłįchǫ can use the evidence to improve and refocus their programming, increase knowledge and continue to improve safe condom use practices.

## Background

Over the past two decades, many Aboriginal communities in Canada have settled long-standing land claims and signed self-governing agreements with federal and territorial governments. New government means new responsibilities and opportunities to generate policies, protocols and services that reflect the needs, values, and culture of their communities.

Despite many well-intentioned health programmes and policies, the burden of illness among rural Aboriginal communities in northern Canada continues to grow [1,2]. Initiatives originating outside the communities have had limited success in reducing outbreaks of preventable disease, prompting communities and their health organizations to seek a different approach.

Established by the new Tłįchǫ Government in 2005, the Tłįchǫ Community Services Agency (TCSA) is part of an Intergovernmental Services Agreement between the Government of Canada, the Government of the Northwest Territories (GNT) and the Tłįchǫ Government. The TCSA delivers services of the *public* territorial government and of the *tribal* (First Nation) Tłįchǫ government [3]. Now in year three of a ten-year transition period, an early step in the transfer was to integrate education, health and social services programmes and professional services to embody traditional community values under the umbrella of TCSA.

**Figure 1:**
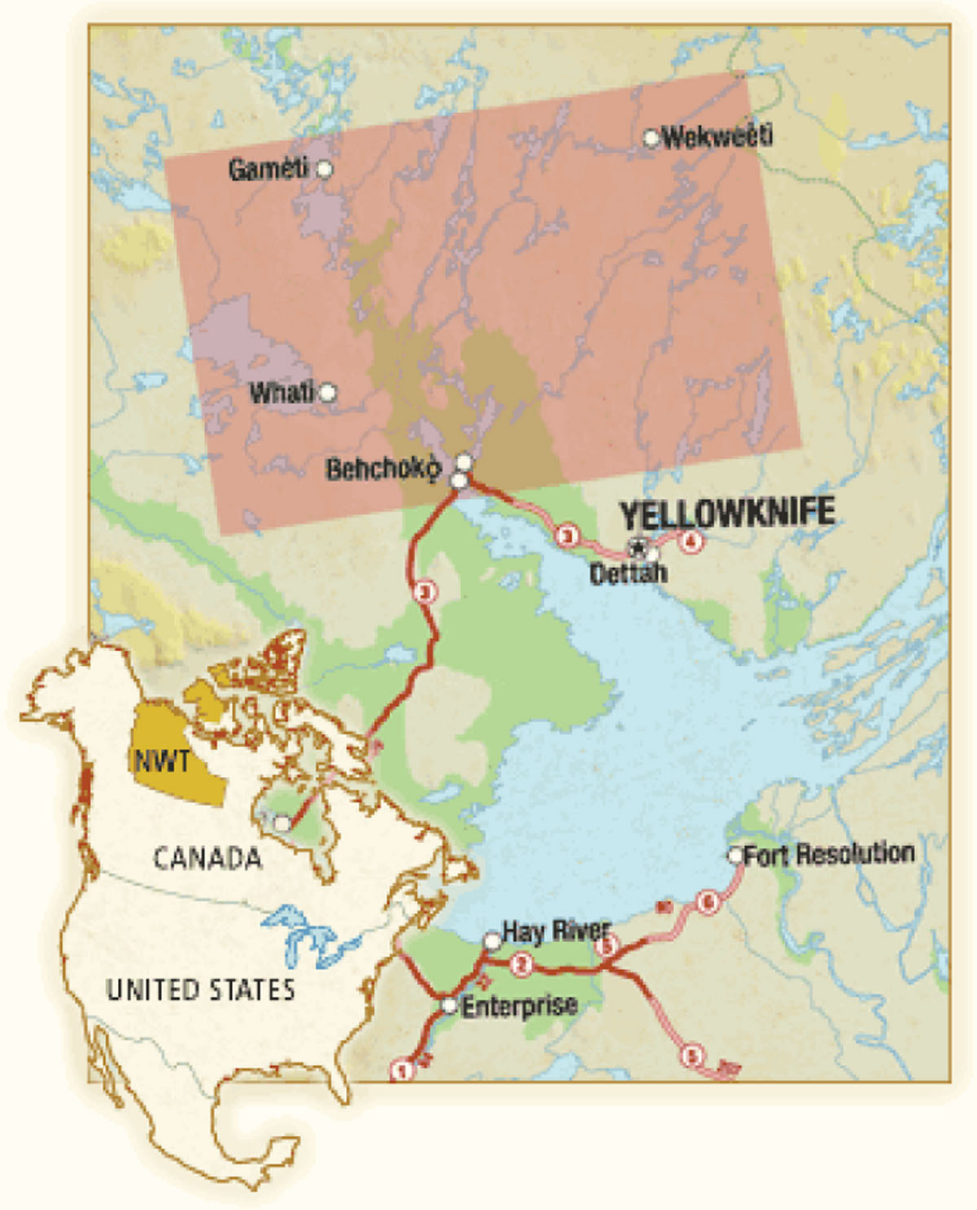
Map of Tłįchǫ region

There are 2,955 residents in the four Tłįchǫ communities (Behchokö, Whatì, Gamètì, and Wekweètì) in the Northwest Territories (NT) of Canada. Behchokö, the region’s administrative centre, is a one-hour drive west of Yellowknife. Gamètì, Wekweètì and Whatì are smaller remote communities accessible only by air in summer and ice road in winter. The population is young, with 31% under the age of 15, and an annual growth of 1%, which challenges regional health and social services [4]. Poor health outcomes like substance abuse, risky sexual behaviours, increased rates of chronic disease, and poor decision-making skills are well documented in relation to intergenerational effects of residential schooling, lack of housing, low employment, and challenges in attaining higher levels of education [5-7]. TCSA acknowledged these challenges by developing a health management approach that incorporates local knowledge that in turn drives local solutions.

TCSA is particularly concerned about rising rates of sexually transmitted infections (STI). August 2008 saw an outbreak of syphilis, a disease that was nearly non-existent over the previous eight years. In 2009, STI rates in the NT were ten times higher than the national rate and Tłįchǫ regional rates were nearly four times that of the NT – 91 cases per 1,000 (unpublished 2009 data, GNT). STI epidemiology in Aboriginal communities requires innovative approaches [8,9]. TCSA incorporates regional perspectives in evidence-based health management to develop culturally safe interventions that address regional risk factors, behaviours, and attitudes [10,11].

Prior to 2006, the TCSA STI programme relied on descriptive statistics provided by the GNT Department of Health and Social Services. These data lacked detail for regional funding priorities, programme goals, and staffing decisions. In order to make informed decisions about programme direction and resource allocation, TCSA and the community-based Healing Wind Advisory Group investigated sexual health knowledge, attitudes and behaviours through a baseline cross-sectional survey in 2006/07 and a follow-up survey in 2010, which informed prevention activities and interventions. TCSA invited CIET to support this research on the local factors that underpin the regional rates [12].

## Methods

### Study design

Economists, consumer relations, and non-profit organizations have used the term *social audit* to refer to social accountability of corporations in the development and distribution of products and/or services [13-15]. CIET uses the term to describe a research method that customizes the design, data collection and analysis by combining qualitative and quantitative approaches and contextualizing research outcomes through community participation and leadership. The aim of social audit is to improve communication and dissemination of results, and optimizes resource allocation [16-20].

A single social audit cycle has two phases; each phase has several activities [21]. The Tłįchǫ completed a full social audit cycle through: 1) design and data collection of the baseline survey to voice regional sexual health attitudes, beliefs, and behaviours; and 2) socialization of baseline survey data for participatory action through evidence-based programming. The follow-up survey marked the beginning of the second social audit cycle that measured the impact of sexual health interventions implemented by the Tłįchǫ Community Action Research Team (CART).

### Ethics

This project is part of CIET’s sexual health research initiative, Aboriginal youth resilience to HIV/AIDS (ACRA), which received ethics approval from the Health Canada Research Ethics Board (REB-2006-0016). The Aurora Research Institute under the GNT issued license number  14932 in 2011. The TCSA developed the Healing Wind Advisory Group made up of regional Elders, Tłįchǫ health care professionals, and regional managers to make sure this research also met the standards of the local Tłįchǫ leadership. They advised on cultural protocols for discussing sensitive topics such as sexual health, the design and wording of questionnaires, target age groups, partnership development, communications, and provided support for the community based researchers.

### Instrument development

The Healing Wind Advisory Group designed the baseline and follow-up questionnaires which identified regional beliefs, knowledge, attitudes and behaviours, use of alcohol and drugs, knowledge, attitudes and beliefs around HIV, STIs and sexual health, access to sexual health information, and Tłįchǫ culture and traditions. Cultural questions included questions about how often they attended religious services, to what degree they followed a Tłįchǫ way of life, and how often they took part in traditional Tłįchǫ practices or ceremonies.

Healing Wind advisers and the community based researchers piloted the questions to enhance clarity, relevance, and cultural appropriateness by testing within the group members and recruiting twenty people representative of the target survey population: 14 years and older, parents, elders, men and women in a variety of sites including party houses (houses frequented by those involved in regular substance abuse and other risky behaviours), schools, work places, and public spaces.

During the baseline questionnaire pilot, Healing Wind identified that the Tłįchǫ terminology about sexuality and sexual health had negative connotations, related to the regional influence of longstanding religious value systems. The original terminology may have biased participant responses. Before the baseline survey, a terminology workshop with Healing Wind language experts identified more neutral terms to decrease potential bias and to develop new ways to discuss sexual health. Local health care professionals used the new terminology in radio programming where they shared the results of the baseline survey. This new terminology is now used in intervention programming.

Community based researchers administered a baseline cross-sectional survey from December 2006 to January 2007 and a follow-up survey in May 2010. The follow-up survey incorporated new questions in response to Healing Wind’s concerns about changing attitudes and practices around forced sex, whether participants frequented party houses, and their experiences with gossiping, bullying, and violence. The survey also explored participation in CART interventions implemented since the baseline.

### Sample and data collection

Community based researchers used a broad recruitment strategy to gain participation of those aged 14 and older. According to the regional health data, the youngest reported case of STI was 14 years. Participants’ ages ranged from 14 to 95 years, providing information on all potentially sexually active age groups. Recruitment targeted residential areas, workplaces, youth centres, community buildings, and party houses. The community based researchers recruited participants, explained how to complete the questionnaire and remained with participants through to completion of the survey. Participants could refuse to answer any question and could stop answering at any point. Community researchers debriefed with each other daily to explore strategies for strengthening consistency and quality control of the survey process.

### Bias

Surveys involving sensitive topics like sexual health can introduce participation and reporting biases [22-24]. Local community based researchers administered both surveys since the team felt they understood sensitive cultural values around sexual health in their communities, and recognised potential selection and information biases [25,26]. They received rigorous training in questionnaire administration to decrease bias and to respect cultural protocols. For confidentiality, community researchers from the smaller communities did not administer surveys in their home communities. Local researchers travelled in pairs and allowed participants to choose the researcher with whom they were most comfortable.

During the 2007 baseline survey, we had difficulties reaching higher risk sub-groups; inclusion of party houses for the follow-up survey helped to rectify this omission, but may have increased reporting of high risk activities. Possible explanations for non-participation in both surveys included absence from the community for employment, school, cultural or medical reasons. Some respondents may have been uncomfortable with the survey content or the large number of questions, and this may have increased non-response or differential responses.

### Data management and analysis

We used the public domain software package EpiInfo [27] to digitise survey data. Double data entry with validation reduced keystroke errors. Analysis relied on open source software CIETmap [28,29], which includes a user-friendly interface to the popular R programming language [30,31]. Population weights matched sample proportions of each community to those of the Tłįchǫ population. We examined associations between factors in bivariate and then multivariate analysis using the Mantel-Haenszel procedure [32,33]. We describe associations using the Odds Ratio (OR) accompanied by the 95% confidence interval (CI).

Analysis focused on the principal outcome of safe condom use behaviours: a composite variable that included condom use with non-regular partners, multiple sexual partners in the past month and condom use the last time they had sex. The target sample for analysis included all participants accessing CART’s education programming created in direct response to feedback from community discussions of the baseline survey findings.

In order to consider whether the intervention did not work at all, or whether it simply did not have time to work, we examined intermediate outcomes using a behaviour change model developed by Andersson and colleagues [34,35]. Identified by the acronym CASCADA, this model extends the conventional knowledge, attitudes, practice (KAP) approach to more intermediate outcomes, steps on the path between knowing condom use is important and the act of actually using a condom. These included **C**onscious knowledge (belief that condoms prevent HIV), **A**ttitudes (thinking it is okay to expect sex without a condom, to have more than one partner at a time, and to swap partners), **S**ubjective norms (friends think it is okay to have sex without a condom), intention to **C**hange (planning to use a condom the next time they had sex), **A**gency (would have sex with their partner even if they refused to use a condom), **D**iscussion (able to openly discuss condom use and sex), and **A**ction (using a condom when they have sex, not having multiple sexual partners).

### Intervention and exposure to the intervention

The principal exposure in our analysis was participation in CART’s evidence-based sexual health communication activities. The team delivered sexual health interventions between the baseline and follow-up surveys. Key baseline survey findings provided a focus or theme for the intervention and local knowledge and community discussions designed and tailored the intervention. From the baseline data, the Healing Wind Advisory Group identified the need to improve knowledge of STIs, change sexual attitudes, and enhance safe condom use behaviours as targets for regional interventions. The CART led community discussions about preferred presentation formats for sexual health educational material and potential condom access issues. The outcomes informed the upgrading of educational material to new audio and visual formats. CART also created a condom distribution program that made condoms available in every public washroom across the region to reduce negative connotations around sexually related activities, enhance discussion, and encourage safe sexual practices.

Due to the variation in the intervention content, length and degree of participant interaction, we examined the associations of CART exposure from three different perspectives: participation in any activity, levels of increasing participation (two or more, three or more activities), and community assigned *a priori* rankings (based on community assessment of their expected impact on condom use behaviours).

The analysis also took account of other variables potentially related to condom use such as age, gender, marital status, self-confidence, ability to make choices, following a Tłįchǫ way of life, and participating in Tłįchǫ practices. It also took account of employment status, getting drunk, going to party houses to use alcohol and drugs, feeling at risk for STIs and the feeling that a partner is at risk for HIV. Being a victim of gossip, being threatened, disrespected or verbally abused, beaten, kicked or slapped by a partner in the past year, being forced to have sex, and the ability to say no to sex were included as well as sources of sexual health information such as radio, pamphlets, posters, the youth centre, the internet, and visits to the health centre.

## Results

### Evidence base

Community based researchers collected data from a total of 1,354 respondents (aged 14 and older) in the baseline survey and 1,034 in the follow-up survey. Approximately one half of the respondents were female in both surveys. Those aged 14 to 50 represented 80% (1,039/1,311) of the baseline participants and 85% (822/964) of the follow-up survey (Table 1).

**Table 1:** Evidence base

	2006/07		2010			
		
	N	Weighted %	n	Weighted %	p-value	χ**^2^**
**Total Respondents**	1354		1034			

Female	742/1335	56	585/1019	57	>0.25	0.79
Aged 14–50	1039/1311	80	822/964	85	<0.001	13.51

### Condom use and other outcomes

Sixty-eight percent (637/935) of follow-up survey participants reported they practiced safe condom use. Condom use during the last sexual encounter increased slightly between the baseline and follow-up survey (Table 2a). The same proportion of baseline (90%) and follow-up participants (91%) *reported* they did not have more than one sexual partner in the past month (Table 2a). Nearly half of follow-up participants indicated they used a condom with their non-regular partner in the last year.

**Table 2:** Principal and intermediate outcomes (all participants): 2006/07-2010

	2006/07		2010			
**2a) Principal outcome**	**n**	**Weighted %**	**n**	**Weighted %**	**p-value**	χ**^2^**

Practiced safe condom use behaviours	N/A	N/A	637/935	68	0.0000	>10.38
*Component variables of safe condom use behaviours*						
Used condom in last sexual encounter	660/1301	51	562/965	58	<0.001	12.57
Used condom with non- regular partner in the last year	N/A	N/A	235/524	45	0.0000	>10.38
Did not have more than one partner over the last month	1183/1307	90	889/977	91	>0.25	0.15

**2b) Intermediate outcomes**						

Believes condoms prevent HIV	597/1321	45	727/939	77	0.0000	234.98
Does not believe it is okay to expect sex without a condom	876/1316	67	647/937	69	>0.25	1.54
Does not feel it is OK to have more than one sexual partner at once	1022/1303	78	811/952	85	<0.001	16.50
Would not have sex with partner if they refused to wear a condom	732/1309	56	550/923	60	0.10	2.98
Did not have sex in exchange for money	1264/1313	96	957/985	97	>0.25	1.16

Among five intermediate outcomes comparable between surveys, belief that condoms prevent HIV showed a 32% increase (Table 2b). There were smaller improvements in attitudes towards having multiple sexual partners and having sex with their partner without condoms. The follow-up survey also included variables of condom use not included in the baseline survey. Nearly 60% (559/939) of follow-up respondents said they planned to use a condom the next time they had sex; 61% percent (575/933) were able to talk openly about condoms; and 47% (440/944) were able to talk openly about sex.

### CART exposure and condom use in high risk situations

Bivariate analysis of the principal outcome and exposure to CART interventions showed those who participated in many individual CART activities were *less* likely to have safe condom use practices (Table 3a). We identified five additional factors associated with safe condom use practices yet none of these variables explained the negative relationship between safe condom use practices and participation in CART interventions.

**Table 3:** Participation in individual CART activities and condom use (all ages) in 2010: bivariate analysis

3a) Safe condom use behaviours				
**CART activity**	**Among participants**	**Among non-participants**	**OR**	**95%CI**

*Assembly booth* – annual 3-day regional event where CART runs a sexual health education booth.	97/159	457/648	0.65	0.45-0.96
*Weekend outreach van* –run during 3-4 high risk weekends / year (i.e. holidays, hand games, etc) from 11PM to 3AM targeting high risk areas / individuals.	49/87	499/712	0.55	0.34-0.91
*Interviews and group discussions* –of baseline results for further input on findings.	68/126	479/673	0.47	0.32-0.72
*Community presentations –* of baseline findings and conversations about sexual health in the community.	98/161	453/641	0.65	0.45-0.95
*Condom distribution program –*makes condoms regularly available in all public washrooms across the region.	74/122	477/678	0.65	0.43-1.00
*Youth safe house-* provides safe place for youth who live in high risk homes during the Christmas holidays.	37/72	511/723	0.44	0.26-0.75

**3b) Condom use during last sexual encounter**				

**CART activity**	**Among participants**	**Among non-participants**	**OR**	**95%CI**

*Weekend outreach van*	60/86	417/734	1.75	1.08-3.11
*Dreamcatchers workshop -* annual conference in Edmonton that CART helps organize	85/122	393/698	1.78	1.18-2.86
*Suicide prevention presentations –* one time event to address immediate suicide issues in the region	86/129	390/691	1.54	1.04-2.40
*Regional youth conference –* annual event that addresses social and health related issues including sexual health	106/156	369/664	1.69	1.17-2.55

* Associations were tested with all of the CART activities including assembly booth, weekend outreach van, interviews and group discussions, community presentations, Dreamcatchers workshop, CART Facebook site, condom distribution program, World AIDS day, suicide prevention workshops, youth safe house, and the regional youth conference.

### CART exposure and condom use during last sexual encounter

We also explored the relationship of participation in individual CART activities with condom use practices the last time the respondent had sex (one of the variables that composed the principal outcome). Table 3b shows the association of CART activities with condom use during the last sexual encounter. Four individual activities also showed a significant association with condom use during the last sexual encounter: *the weekend outreach van* (OR 1.75, 95%CI 1.08-3.11, 60/86 among those who participated in the van programme, 417/734 among those who did not participate); *Dreamcatchers workshop* (OR 1.78, 95%CI 1.18-2.86; 85/122 among those exposed to Dreamcatchers, 393/662 among those not exposed); *suicide prevention presentations* (OR 1.54, 95%CI 1.04-2.40; 86/129 among those exposed to suicide prevention presentations, 390/691 among those not exposed); and *the regional youth conference* (OR 1.69, 95%CIa 1.17-2.55; 106/156 among those exposed to the regional youth conference, 369/664 among those not exposed) (Table 3b). Bivariate analysis showed associations of condom use during the last sexual encounter with nine additional variables, beyond CART participation, related to safe condom use and practices (Table 4).

**Table 4:** Unadjusted bivariate analysis of related variables and condom use during last sexual encounter (all ages) in 2010

Factor	Condom use during last sexual encounter among those with factor	Condom use during last sexual encounter among those without factor	OR	95%CI
Aged 14-50	472/780	56/128	1.97	1.33-2.96
From Behchokö	312/539	250/425	0.96	0.74-1.26
Male	296/408	286/546	1.76	1.34-2.32
In a steady relationship	147/443	407/511	0.13	0.09-0.17
Grade 9 or higher	388/684	170/275	0.81	0.60-1.09
Participates in a Tłįchǫ way of life	508/847	42/91	1.75	1.11-2.80
Regularly participates in Tłįchǫ ceremonies	175/269	373/667	1.47	1.09-2.01
Does not drink	161/298	387/642	0.77	0.58-1.03
Does not use street drugs	410/753	142/198	0.47	0.32-0.66
Did not go to party houses to use drugs and alcohol	324/601	227/346	0.61	0.46-0.81
Has not been a victim of gossip	346/596	210/353	0.94	0.71-1.24
Has not been threatened, disrespected or verbally abused in past year	401/662	146/278	1.39	1.04-1.86
Has not been beaten, kicked or slapped in the past year	446/768	101/174	1.00	0.70-1.41
Has not been forced to have sex	423/719	86/162	1.26	0.88-1.81
Able to refuse sex with their partner	333/611	203/310	0.63	0.47-0.84
Does not feel their partner is at risk for STIs	319/560	212/360	0.92	0.70-1.22
Does not feel they are at risk for STIs	244/429	318/536	0.90	0.69-1.18

Regarding exposure to at least one CART activity (potential yes/no response) there was a considerable amount of missing data on CART participation (226 missing out of 1034). Among those who responded, 39% (315/808) had participated in at least one of the 11 different activities. If a participant left any of the CART activity questions unanswered we coded their missing responses as if they had not participated in the activity. Taking into consideration all participants who said “yes” or “no” to exposure, the association with condom use during the last sexual encounter strengthened with increasing participation in CART activities (Table 5a). Those who participated in three or more CART activities were almost twice as likely to use a condom during the last sexual encounter than those who did not participate in three or more interventions (OR 1.48, 95%CI 1.03-2.21; 101/153 among those exposed to three or more CART interventions, 461/812 among those not exposed) (Table 5a). In turn, exposure to at least one of the community assigned *a priori* highest-ranking activities was similarly associated with condom use during the last sexual encounter (OR 1.40, 95%CI 1.06-1.86; 209/329 among those who participated in at least one top ranked activity, 353/636 among those not exposed) (Table 5b).

**Table 5:** Participation in CART activities and condom use during last sexual encounter (all ages) in 2010: bivariate analysis

	Condom use during last sexual encounter among exposed	Condom use during last sexual encounter among non-exposed	OR	95%CI
**5a) Increasing participation in CART activities**				

Exposed to at least one activity	195/308	367/657	1.36	1.03-1.83
Exposed to two or more activities	132/202	430/763	1.46	1.05-2.07
Exposed to three or more activities	101/153	461/812	1.48	1.03-2.21

5b) Participation in at least one top ranked CART activity*				

Exposed to at least one top ranked activity	209/329	353/636	1.40	1.06-1.86

*Team members ranked each individual intervention activity based on their collective assessment of the expected impact of the activity. The highest ranked activities were considered the CART activities that would have the most impact on behaviour change based on their targeted content and length of exposure. These highest ranked activities included the Assembly Booth, weekend outreach van, interviews and discussions, Facebook, condom distribution program, youth conference.

Multivariate analysis of condom use during the last sexual encounter examined associations with participation in three or more CART interventions and the nine additional variables associated with condom use in bivariate analysis. The final model of the multivariate analysis identified a steady relationship as a strong predictor of not using condoms (OR 0.13, 95% CI 0.10-0.17; 147/443 among those in a steady relationship, 407/511 among those not in a steady relationship used a condom on last sexual encounter). The multivariate analysis also revealed that those who participated in at least one of the top ranked CART interventions were one and a half times as likely to use a condom the last time they had sex (Table 6).

**Table 6:** Multivariate model of CART participation (at least one top ranked activity) and condom use (all ages): 2010

*Outcome: used a condom the last time they had sex* N=*954*	OR	95%CI
Participated in at least one a priori ranked CART activities	1.45	1.07-1.98
In a steady relationship	0.13	0.10-0.17

### CART exposure and intermediate outcomes

Table 7 shows bivariate analysis of exposure to CART activities with the intermediate outcomes considered in our CASCADA model of behaviour change. Participation in three or more activities was associated with positive intentions around condom use, ability to talk about sex and condoms as well as whether participants had more than one sexual partner in the past month (Table 7a). However, exposure to at least one of the *a priori* highest ranked CART activities was only associated with intention to use a condom the next time they had sex (Table 7b). Additional analysis was unable to explain the associations for any of the intermediate outcomes to any degree of participating in CART interventions.

**Table 7:** Associations between participation in CART activities and intermediate outcomes (all ages) in 2010: bivariate analysis

7a) Exposed to three or more activities	Among those exposed	Among those not exposed	OR	95%CI
Plans to use a condom the next time they have sex	109/154	450/785	1.80	1.24-2.75
Can openly talk with others about condoms	114/152	461/781	2.08	1.41-3.28
Can talk openly about sex	85/154	355/790	1.51	1.05-2.19
Has not had sex with more than one person in the last month	131/154	758/823	0.49	0.29-0.90

**7b) Exposed to at least one top ranked activity**	**Among those exposed**	**Among those not exposed**	**OR**	**95%CI**

Plans to use a condom the next time they have sex	215/325	344/614	1.53	1.15-2.06

## Discussion

Studies of sexual health management in indigenous communities generally focus on health care professional practice [36] and treatment [37]; few studies examine the impact of community-driven sexual health prevention interventions [38]. Our study does not show a positive change in our principal outcome of safe condom use practices, which was a composite outcome including condom use with non-regular partners and having multiple sexual partners. The target population for CART activities also included the higher risk population who may have had higher risk condom practices. We believe a differential reporting bias [39] may also have affected the measurement, with disclosure rates about non-regular partners increased by exposure to CART. The interventions were associated with ease in discussing condom use and sex, which may have made CART-exposed participants more comfortable with reporting these types of risky behaviours. Harnak and colleagues found similar biases in a food intake study where the study intervention served as a predictor of reported food intake [40]. Also, a reporting bias may occur if participants are reluctant to respond truthfully to questions that hold stigma in the region such as ‘did you have more than one sexual partner in the last month’ and ‘did you use a condom with your non-regular partner in the last month’. Responding to a less judgmental question such as ‘did you use a condom the last time you had sex’ may be easier to answer honestly.

Condom use during the last sexual encounter, however, showed a significant shift to safer condom use practices, strongest in the interventions the community team anticipated would have the most impact. Increasing exposure to CART activities was associated with condom use during the last sexual encounter. Study outcomes will help the CART to tailor their current condom related programmes, target areas for new development, and in turn hopefully affect further behaviour change. Community partners can enhance decision making in all stages of analysis and targeted evaluation of intervention programming.

We also looked at intermediate outcomes for evidence of additional impact. Participation in three or more CART activities was associated with discussion of condoms and sex with other people. CART discussions of condom use in focus groups of high-risk people helped to define why condom use was low across the region. Discussion is the last step in the CASCADA behaviour change model before action: using a condom. This suggests refined programming may improve condom use even more.

Other studies support the transition between a respondents’ discussion of behaviours and change in behaviour itself. In a ten-country study, Mitchell and colleagues found that respondents who said they talked about HIV were more likely to report being tested for HIV in the preceding 12 months [41]. Likewise, Cockcroft and colleagues found similar associations between parental discussion of vaccination and the likelihood of childhood vaccination [18].

Our analysis examined how people who participated in evidence-based community driven interventions changed their condom use behaviours compared with those who did not participate in the interventions. We took into account other factors related to condom use such as age, gender, cultural practices, sexual health attitudes, and violence. We also looked at intermediate outcomes such as attitudes around condoms and risky sexual behaviours, ability to discuss sex and condom use, and multiple sexual partners. The study applied a mixed methods approach parsed, in the linguistic sense, into different ‘moments’ (project design, instrument development, data collection, data entry and cleaning, analysis, interpretation, and dissemination) that required quantitative or qualitative methods depending on the research phase [42]. We were able to draw a line between different moments in the research process, stepping between naturalistic designs to a positivistic data collection approach, from mechanical data entry and cleaning to participatory analysis and interpretation, which in turn produced a series of community-led interventions [21].

### Study limitations

It is never easy to balance scientific rigour with community engagement and ownership in research [43]. Partin observed that “without responsiveness, the partnerships needed to successfully develop and implement community-based programmes either may never form or may disintegrate over time, and without rigour the programmes resulting from these partnerships may be doomed to failure [44].” Building community engagement and leadership into epidemiological studies can be challenging, yet is essential to inform research design, methods, and interpretation of results.

The community’s decision to conduct the follow-up survey at a time when more community members were away for schooling or regional events probably influenced recruitment and produced a smaller sample size than anticipated. Even though community based researchers explained the proper way to complete survey questions, the questionnaire format, participant literacy or concentration levels resulted in too much missing data; respondents indicated they had not participated in the first two CART activities listed and then left the remaining nine CART activities blank. The questions related to CART participation might not have been sensitive enough to detect the impact of each activity on its own.

We see these weaknesses as the challenge of translating high quality research into protocols and training for community-level evidence-based planning.

## Conclusion

The Tłįchǫ experience illustrates how naturalistic and positivistic approaches can compliment and reinforce one another in social audit. The shifts in various CASCADA outcomes may be early indicators that CART is affecting small changes in how Tłįchǫ communities perceive sexual health, even though we could not confirm an impact on condom use behaviour. These study outcomes will help the Tłįchǫ to tailor their current condom related programmes and to target areas for new development. Subsequent follow-up studies will add to the existing evidence-base and help to build the community voice in Tłįchǫ planning and management.

## List of abbreviations

CIET: Community Information, and Epidemiological Technologies; TCSA: Tłįchǫ Community Services Agency; NT: Northwest Territories; CART: Community Action Research Team; GNT: Government of the Northwest Territories

## Competing interests

The authors declare that they have no competing interests.

## Authors' contributions

**KE** contributed to instrument design, conducted data analysis and drafted the manuscript. Her contribution to this article was part of her doctoral studies at the Universidad Autónoma de Guerrero in Mexico. **NG & JM** contributed to instrument design, developing methods, drafting of the manuscript and guidance for community leadership throughout the research. **NA** and **SM** provided input and guidance for study design, data analysis and interpretation.
